# Facile Fabrication of Platinum-Cobalt Alloy Nanoparticles with Enhanced Electrocatalytic Activity for a Methanol Oxidation Reaction

**DOI:** 10.1038/srep45555

**Published:** 2017-03-30

**Authors:** Huihong Huang, Xiulan Hu, Jianbo Zhang, Nan Su, JieXu Cheng

**Affiliations:** 1 College of Materials Science and Engineering, Nanjing Tech University, Xin-Mo-Fan Road No. 5, 210009, Nanjing, Jiangsu, China; 2 The Synergetic Innovation Center for Advanced Material, y, Xin-Mo-Fan Road No. 5, 210009, Nanjing, Jiangsu, China; 3 Jiangsu National Synergetic Innovation Center for Advanced Materials (SICAM), Xin-Mo-Fan Road No. 5, 210009, Nanjing, Jiangsu, China

## Abstract

Decreasing the cost associated with platinum-based catalysts along with improving their catalytic properties is a major challenge for commercial direct methanol fuel cells. In this work, a simple and facile strategy was developed for the more efficient preparation of multi-walled carbon nanotube (MWCNT) -supported Pt/CoPt composite nanoparticles (NPs) via solution plasma sputtering with subsequent thermal annealing. Quite different from general wet synthesis methods, Pt/CoPt composite NPs were directly derived from metal wire electrodes without any additions. The obtained Pt/CoPt/MWCNTs composite catalysts exhibited tremendous improvement in the electro-oxidation of methanol in acidic media with mass activities of 1719 mA mg^−1^_Pt_. This value is much higher than that of previous reports of Pt-Co alloy and commercial Pt/C (3.16 times) because of the many active sites and clean surface of the catalysts. The catalysts showed good stability due to the special synergistic effects of the CoPt alloy. Pt/CoPt/MWCNTs can be used as a promising catalyst for direct methanol fuel cells. In addition, this solution plasma sputtering-assisted synthesis method introduces a general and feasible route for the synthesis of binary alloys.

Direct methanol fuel cells (DMFCs) have attracted extensive attention as a promising potential sustainable power source due to their clean emissions, high power density and high energy conversion efficiency^1^,^2^,^3^. Platinum is the most significant catalyst for DMFCs due to its exceptional catalytic performance. However, single-component Pt NPs suffer from high cost, poor stability and low resistance to CO poisoning for the methanol oxidation reaction (MOR)^4^,^5^,^6^. Hence, many efforts have been devoted to reducing the cost and enhancing the catalytic activity and long-term stability of Pt-based catalysts for the MOR.

Alloying Pt with cheaper metals (Co, Cu, Ni and Fe) is an effective approach for tailoring the electronic and geometric heterostructures to improve activity and stability^7^,^8^,^9^,^10^,^11^. Compared with various Pt bimetallic alloys (with Cu, Ni or Fe), the Pt-Co alloys are generally more stable due to the higher degree of alloying of cobalt and platinum^3^,^12^. Furthermore, cobalt atoms embedded into the Pt lattice give rise to compressive strain in the lattice and narrow the distance of the Pt-Pt bond. Impressively, the different electronegativity values (Pt is 2.28 and Co is 1.88) result in filling the Pt d-band and downshifting of d-band centre. Thereby, more active sites of the catalysts are exposed while weakening the binding of oxygenated species (-OH and -OOH) due to the electronic charge transfer from Co to Pt^13^,^14^,^15^,^16^,^17^. Therefore, the catalytic performance of Pt-Co alloys are enhanced with their special synergistic effects. For example, three-dimensional Pt-Co alloy networks showed high activity and high stability ascribed to their structure stability and synergistic effects^18^. Furthermore, electronic modification by Co introduced into Pt_3_Co, Pt-Co nanowire assemblies exhibited superior CO tolerant behaviour compared to Pt NPs^15^. Thus, the efficient synthesis of high-quality, uniformly dispersed Pt-Co alloy NPs is a promising strategy for developing advanced hybrid catalysts in DMFCs.

Until now, various physical and chemical synthesis methods of Pt-based alloy NPs have been surveyed, including incipient wetness^19^, wet impregnation^20^,^21^,^22^, the polyol method^23^,^24^,^25^,^26^, electrochemical deposition^27^,^28^,^29^, the colloidal method^30^,^31^,^32^, and chemical vapor deposition (CVD)^16^,^33^,^34^. However, these methods are subject to intrinsic drawbacks, such as (a) complicated multistep processes, including mixing, precipitation, and cleaning; (b) catalyst poisoning by chemical residues and lack of a “clean” surface; (c) strict conditions required for alloying; and (d) high vacuum sputtering and advanced patterning equipment, similar to the CVD process.

Solution plasma sputtering is a simple and facile technique for the synthesis of NPs in a solution and consists of the following processes: (a) the bombardment of rapid energetic radicals, (b) the fast diffusion of atomic vapor, (c) plasma expansion, and (d) solution medium condensation^35^,^36^. Carbon-supported PtAu^37^ and PdAu^38^ alloy NPs with diameters of approximately 2–5 nm have been fabricated directly through this technique in our previous work. The as-obtained alloy NPs are directly derived from their metal electrodes without any additions in an open system under atmospheric pressure. In addition, this technique is suitable for the preparation of highly dispersed nanomaterials on a variety of carriers, such as metal oxides and carbon materials. Carbon nanotubes (CNTs) have been used to support bimetallic NPs as a promising electro-catalyst for the MOR, owing to their higher electrical conductivity, higher catalyst loading efficiency, higher stability and higher surface-to-volume ratio compared with carbon black^12^,^39^.

In this work, a simple and facile route for more efficient fabrication of MWCNT-supported Pt/CoPt composite NPs is reported, achieved by solution plasma sputtering with subsequent thermal annealing from metal wire electrodes. As-obtained Pt/CoPt composite catalysts exhibited excellent electro-catalytic activity and stability for the MOR due to their high dispersion, the high alloying degree and the “clean” surface of the catalysts.

## Results

### Synthesis of MWCNT-supported Pt/CoPt composite NPs

CNT-supported Pt/CoPt composite NPs with high electro-catalytic activities were synthesized in two steps. For the first step, the schematic diagram of the preparation processes for producing Pt/CoPt composite NPs by a solution plasma technique is shown in Figure S1. In a typical first step of the synthesis of Pt/CoPt composite NPs, the cobalt and platinum wires (Φ = 1.0 mm) were used as opposite electrodes immersed into deionized water without any additions. The gap between the two opposite electrodes was maintained at approximately 0.3–0.5 mm by a screw micrometre during discharge. The discharge was generated using a high voltage pulsed DC power supply (repetition frequency: 15–25 kHz, pulse width: 2–3 μs, Kurita Co. Ltd., Japan) without an inert gas. The colour of the water gradually changed from being colourless to dark grey with increasing discharge time. The amount of metallic hybrid NPs (including Pt, Co and CoPt alloy) was controlled by adjusting the discharge conditions, including voltage, pulse width and time. The consumption of platinum and cobalt wires were 10.1 mg and 2.9 mg, respectively, after discharge for 30 min with the experimental parameters of 2 kV, 20 kHz and 2.5 μs. Immediately following this procedure, the above obtained NPs in uniform suspension were mixed with a dispersed aqueous MWCNT by ultrasonic treatment to prevent NP aggregation and improve their electrical conductivity. Then, the initial composite catalysts were dried at 60 °C in air, denoted as Pt/CoPt-1/MWCNTs. In the second step, Pt/CoPt-1/MWCNTs were annealed at 700 °C for 2 h under flowing N_2_ to obtain Pt/CoPt-2/MWCNTs to obtain a high degree of alloying in the Pt-Co alloy NPs. Here, the mass ratio of Pt/Co was almost fixed to 3.48, and 15 wt% hybrid NPs, including Pt and CoPt, were supported on the MWCNTs. Fe, Ni and Cu wires instead of Co wire served as the respective electrodes; their hybrid alloy nanoparticles were synthesized by a similar process as well.

**Figure 1:**
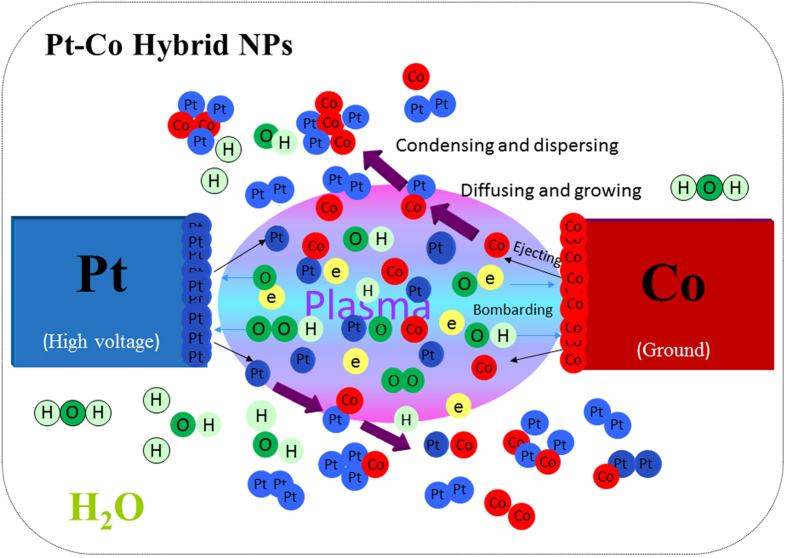
Schematic diagram for the synthesis of Pt/CoPt-1 composite NPs by solution plasma sputtering.

Our facile solution plasma sputtering-assisted synthesis method is adept at the disposition of ultra-small metallic NPs, specifically for multi-metallic systems. In the case of Pt/CoPt composite NPs, the initial fabrication model is illustrated in Fig. 1. Along with the bombardment of highly energetic radical particles, platinum and cobalt atoms were ejected into the plasma region from the electrode pair’s tip, with the plasma expanded in water due to the enormous difference in the temperature and pressure between the plasma and the surrounding water medium. The expanded plasma particles were quickly condensed because of collisions with the relatively low-temperature surrounding molecules. Then, the plasma lost its expansive driving force, resulting in the formation of ultra-small metallic hybrid NPs (including Pt, Co and CoPt alloy) with highly clean surfaces.

### Characterization of MWCNT-supported Pt/CoPt composite NPs

**Figure 2:**
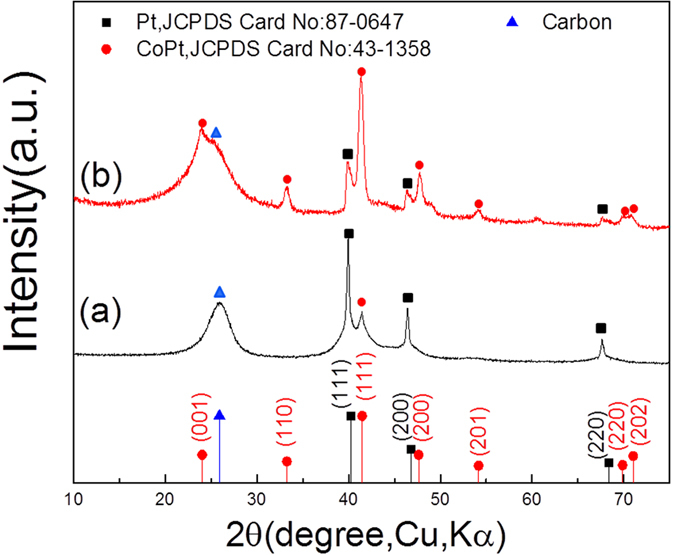
XRD pattern of (**a**) Pt/CoPt-1/MWCNTs and (**b**) Pt/CoPt-2/MWCNTs.

XRD pattern in Fig. 2 confirmed that MWCNT-supported Pt/CoPt composite NPs were prepared directly by solution plasma sputtering and subsequent thermal annealing. Diffraction peaks of both Pt and CoPt were detected. Pt (111), (200) and (220) crystal planes corresponded to the standard card of Pt with a face-centred cubic structure (JCPDS card No. 87–0647, space group Fm-33m (225)). Only one weak peak of the CoPt (111) planes was detected just by solution plasma sputtering, as shown in Fig. 2a. In contrast, seven characteristic diffractions peaks became obvious and corresponded to the typical (001), (110), (111), (200), (201), (220) and (202) planes of CoPt with a face-centred tetragonal structure (JCPDS card No. 43-1358, space group P4/mm (123)) after thermal annealing, as shown in Fig. 2b. It is worth noting that the densities of diffraction peaks of Pt (111), (200) and (220) crystal planes decreased after the subsequent thermal annealing. In addition, the absence of diffraction peaks typical for metal cobalt and cobalt oxides may be attributed to Co being dissolved into the Pt lattice and forming a Pt-Co alloy or being present as an amorphous phase. The alloying temperature of CoPt NPs was approximately 280 °C, as shown in Figure S2. These results clarified that subsequent thermal annealing above 300 °C improved the formation of CoPt alloy NPs and the composite catalyst of Pt/CoPt-2/MWCNTs was composed of the CoPt alloy and small amount of Pt NPs on the MWCNTs.

The chemical composition of Pt and Co loaded on the MWCNT was determined by inductively coupled plasma mass spectrometry (ICP-MS) (shown in Figure S3). Before and after heat treatment, the mass of Pt did not change. The Pt and Co mass ratio of Pt/CoPt-1/MWCNTs was approximately 3.53, in line with the consumption ratio (3.48) of the Pt and Co wires in the discharge process. The Pt and Co compositions of Pt/CoPt-2/MWCNTs changed, the mass ratio and the atomic ratio of Pt and Co were 4.96 and 1.82, respectively. This change may be due to the mass loss of Co during the heat treatment process due to different features of metallic NPs.

**Figure 3:**
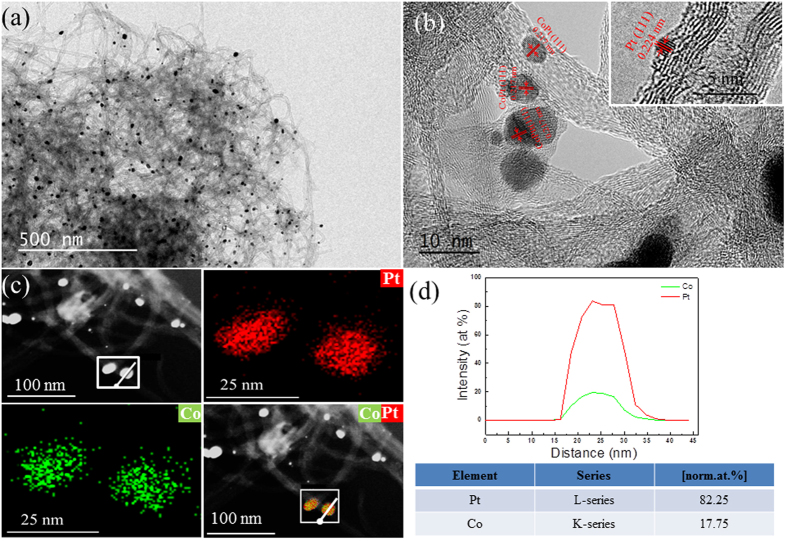
(**a**) TEM and (**b**) HR-TEM images of Pt/CoPt-2/MWCNTs. The inset shows the HR-TEM image of Pt NP. (**c**) HAADF image and EDS mapping of Pt, Co and the composite of Pt and Co. (**d**) Line and area profiles of Pt and Co extracted from the white line and box indicated in (**c**).

Figure 3a shows the highly dispersed Pt/CoPt-2 composite NPs loaded on MWCNTs obtained by solution plasma sputtering-assisted synthesis method. High-resolution TEM (HR-TEM) image of Pt/CoPt-2/MWCNTs (Fig. 3b) shows that the CoPt NPs have well-defined and uniformly spaced lattice fringes with the fringe distance measured to be 0.217 nm, corresponding to the (111) interplanar spacing in the CoPt alloy structure. The inset of Fig. 3b shows monodispersed NPs 2 nm in size with a fringe distance value of 0.224 nm, which agrees with the (111) interplanar spacing of Pt. The results from both HR-TEM and XRD analyses indicate that CoPt NPs have a solid solution structure. The alloy structure is further characterized by the high-angle annular dark field (HAADF) image and energy dispersive X-ray spectroscopy (EDS). Figure 3c shows an HAADF image and the EDS mapping of Pt/CoPt-2/MWCNTs, demonstrating the homogenous distribution of both Pt and Co atoms in the alloyed NPs. For clearer mapping of the atomic position in a CoPt nanoparticle, a line scan profile and an area scan analysis were performed, which are marked with a white line and box in Fig. 3c. As shown in Fig. 3d, the analysis shows an atomic ratio of Pt to Co of approximately 4.63, in agreement with the results of ICP-MS, and the CoPt NPs display an egg-like shape with an average size of 15 nm ± 1 nm.

**Figure 4:**
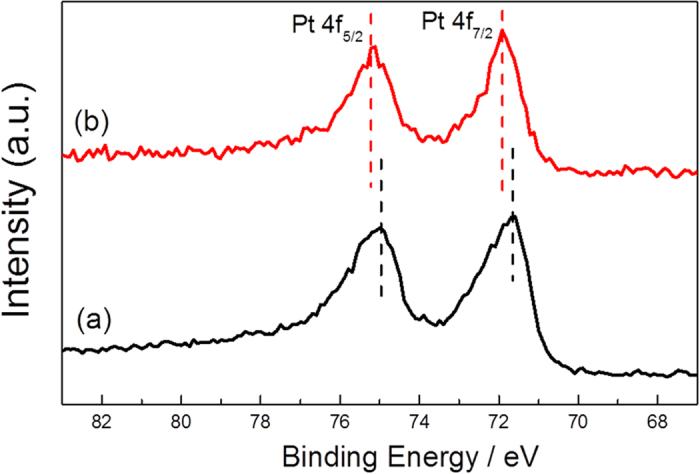
XPS spectra of (**a**) pure Pt/MWCNTs and (**b**) Pt/CoPt-2/MWCNTs in the Pt 4f region.

The alloying of Pt with Co can lead to a change of the electronic structure of Pt due to lattice strain and the charge transfer effect^16^,^40^. To identify the electronic structure of Pt/CoPt-2/MWCNTs, the XPS spectra were obtained. Figure 4 shows the Pt 4f spectrum of Pt/CoPt-2/MWCNTs and pure Pt/MWCNTs. The binding energies of Pt 4f for Pt/CoPt-2/MWCNTs and pure Pt/MWCNTs are 71.92 eV & 71.70 eV (Pt 4f7/2) and 75.21 eV & 74.99 eV (Pt 4f5/2), respectively. The Pt 4f binding energies for Pt/CoPt-2/MWCNTs exhibit a positive shift (0.22 eV) relative to the pure Pt/MWCNTs, indicating that Pt-Co bimetallic alloy NPs were obtained by our facile solution plasma sputtering-assisted synthesis method. Furthermore, the positively shifted binding energy of Pt suggests a downshift in the d-band centre^15^, which would lead to a weak chemical interaction between oxygenated species and the electro-catalytic surface. In addition, the percentage of Pt^0^ in Pt/CoPt-2/MWCNTs is obviously higher than that of pure Pt/MWCNTs by measuring the relative peak areas. Due to the decreased electron back-donation from the Pt surface to the 2π* orbital of CO, the interaction between Pt and Co could also decrease the adsorption of the CO-like intermediate on the Pt surface, and further effectively enhance the electro-catalytic activity and stability for the MOR, in agreement with previous reports^41^.

### Electrocatalytic performance of MWCNT-supported Pt/CoPt composite NPs

**Figure 5:**
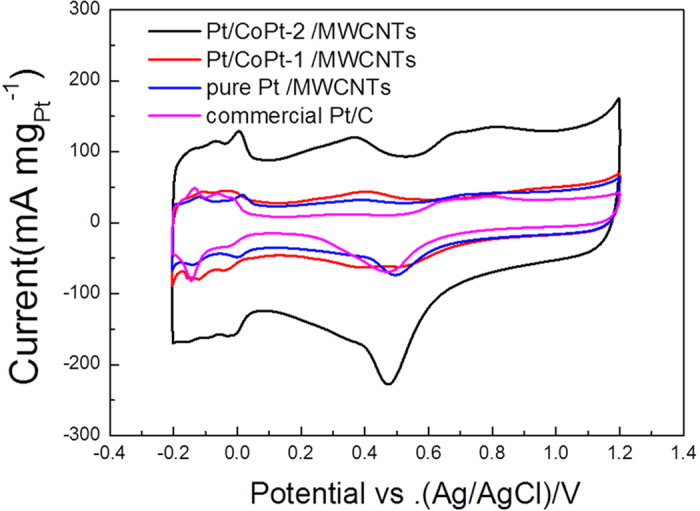
Cyclic voltammograms of Pt/CoPt-2/MWCNTs, Pt/CoPt-1/MWCNTs, pure Pt/MWCNTs and commercial Pt/C measured in argon-purged 0.5 M H_2_SO_4_ at a scan rate of 50 mV s^−1^.

As shown in Fig. 5, the electrochemical active surface area (ECSA) of Pt/CoPt-2/MWCNTs (15 wt%), Pt/CoPt-1/MWCNTs (15 wt%), pure Pt/MWCNTs (15 wt%) and commercial Pt/C (Alfa Aesar, Vulcan XC-72 supported 20 wt% Pt with average diameter of 2 nm, see Figure S4) were evaluated by cyclic voltammetry in argon-purged 0.5 M H_2_SO_4_ at a scan rate of 50 mV s^−1^, in which the current densities are normalized by the mass of Pt. Note that the Pt/CoPt-2/MWCNTs sample has a very high double layer contribution compared to the other samples considering the current value at approximately 0.1 V, which may be due to heat treatment resulting in the good electrical conductivity of the MWCNTs. The specific ECSA was calculated by measuring the charge associated with the hydrogen adsorption/desorption (HAD) potential region after double-layer correction. The ECSA values of the as-prepared catalysts can be easily calculated via the equation Eq. 1, below, with Coulombic charges accumulated during HAD after correcting for the double-layer charging current from the CVs:


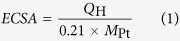


where Q_H_ (millicoulomb, mC) represents the charge due to the hydrogen adsorption/desorption in the hydrogen region of the CV curves. The correlation constant of 0.21 (mC cm^−2^) represents the charge required to oxidize a monolayer of hydrogen on a smooth Pt surface. M_Pt_ (mg) represents the loading of the Pt metal on the working electrode. The ECSA of Pt/CoPt-2/MWCNTs (83.7 m^2^g^−1^) is much larger than that of pure Pt/MWCNTs (27.3 m^2^g^−1^) and commercial Pt/C (41.4 m^2^g^−1^). The larger ECSA was attributed to alloy formation by the solution plasma sputtering technique without any additions and holding many active sites and clean surfaces of catalysts. In addition, the ECSA of Pt/CoPt-2/MWCNTs is obviously larger than that of Pt/CoPt-1/MWCNTs (25.7 m^2^g^−1^) due to the formation of CoPt alloy NPs through the heat treatment at 700 °C for 2 h in N_2_. The formation of the Pt-Co bond is beneficial to downshift the d-band centre of Pt, thus exposing more active sites of Pt and enhancing the electrochemical activities of Pt/CoPt-2/MWCNTs. Therefore, the largest ECSA of Pt/CoPt-2/MWCNTs implied an excellent performance for the MOR.

**Figure 6:**
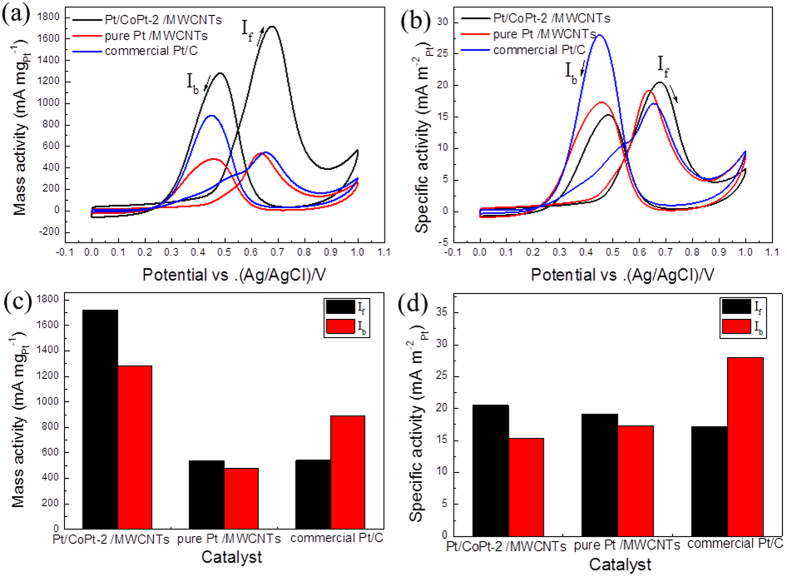
(**a**) Mass activity and (**b**) specific activity of the three catalysts towards methanol oxidation along with respective bar graphs (**c** and **d**) measured in 0.5 M H_2_SO_4_ and 1 M MeOH at a scan rate of 50 mV s^−1^.

Figure 5 clearly indicates that Pt/CoPt-2/MWCNTs show more active sites for methanol electro-oxidation. Figure 6 shows the electro-catalytic activities of Pt/CoPt-2/MWCNTs compared with Pt/MWCNTs and commercial Pt/C towards methanol oxidation were evaluated by cyclic voltammetry in 0.5 M H_2_SO_4_ and 1 M MeOH at a scan rate of 50 mV s^−1^. As shown in Fig. 6a, the mass normalized current density of Pt/CoPt-2/MWCNTs in the positive direction sweep (1719 mA mg^−1^_Pt_) was 3.28 times higher than that of pure Pt/MWCNTs (524 mA mg^−1^_Pt_) and 3.16 times higher than that of commercial Pt/C (543 mA mg^−1^_Pt_). Moreover, the surface area normalized current density of composite catalysts (20.51 mA m^−2^_Pt_) was 1.07 times and 1.20 times higher than that of pure Pt/MWCNTs (19.23 mA m^−2^_Pt_) and commercial Pt/C (17.13 mA m^−2^_Pt_), respectively, as shown in Fig. 6b. Furthermore, Fig. 6c and d show clear differences in the electro-oxidation activities of the three catalysts by the forward peak current density (I_f_) and the back forward peak current density (I_b_). This improved CO tolerance could be attributable to the change in electronic structure (suppression of d-band centre and Pt-Pt distance) of Pt after alloy formation. The possible bifunctional methanol oxidation mechanism for the MOR activity of the Pt-Co alloy catalysts has been explained in previous reports^41^,^42^. Co favourably breaks the water molecule to form Co-OH, while Pt catalyses the methanol dehydrogenation to form Pt-CO instead of forming Pt-OH due to the presence of Co. Consequently, the reaction between Pt-CO and Co-OH produces CO_2_, and the metal surface refreshes for further reaction, leading to enhancement in the activity compared with pure Pt.

Several important reports referring to the catalytic activity towards methanol electro-oxidation are summarized in comparison with our results in Table 1. Therefore, Pt/CoPt-2/MWCNTs exhibit better catalytic efficiency compared with pure Pt/MWCNTs and commercial Pt/C. It is worth noting that Pt/CoPt-2/MWCNTs show remarkably enhanced activity compared with not only commercial Pt/C but also other Pt-Co catalysts.

The stability of electrocatalytic materials has been recognized as one of the most important issues to be addressed before the commercialization of DMFCs. To evaluate the electrochemical stability of Pt/CoPt-2/MWCNTs, chronoamperometric measurements for the MOR were performed in 0.5 M H_2_SO_4_ and 1 M MeOH at 0.6 V. As shown in Figure S5, compared with rapidly decaying current densities of the other two catalysts, Pt/CoPt-2/MWCNTs shows a slower decay at the initial stage of intermediate species (such as CO_ads_) adsorption during methanol oxidation. After several minutes, the current of all catalysts began decreasing slowly, and a pseudo-steady was achieved. The slowest rate of decay of Pt/CoPt-2/MWCNTs indicates their superior CO tolerance compared to pure Pt/MWCNTs and commercial Pt/C, which may be ascribed to the effective CO removal on the surface of the Pt-Co catalysts. This is due to the synergistic effect of the bifunctional mechanism, weakening the Pt-CO bond and promoting the oxidation of CO to CO_2_ via the activation of water in an adjacent site^41^.

**Figure 7:**
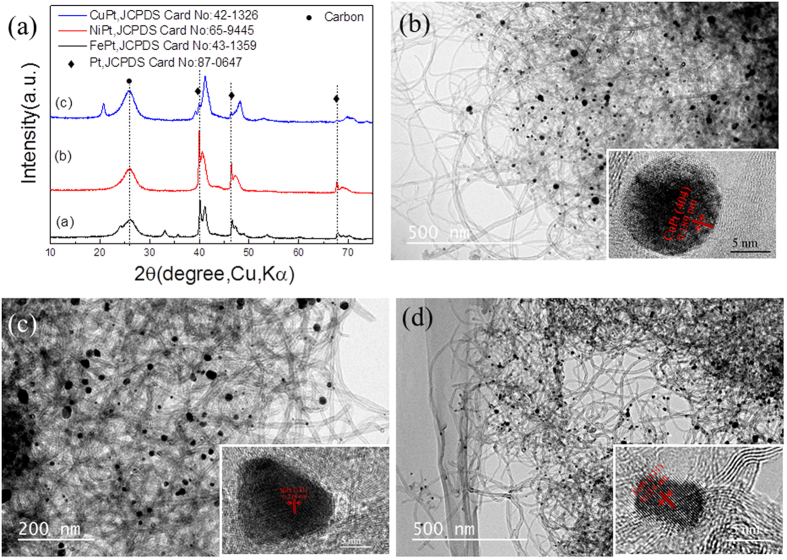
(**a**) XRD patterns and (**b**~**d**) TEM images of Pt/MWCNTs with different alloys of metal and Pt. (**b**) Pt/CuPt/MWCNTs, (**c**) Pt/NiPt/MWCNTs and (**d**) Pt/FePt/MWCNTs.

Pt/M (M = Cu, Ni and Fe) Pt/MWCNTs were also synthesized successfully by this facile solution plasma sputtering-assisted synthesis method, as shown in Fig. 7. The synthesis process was similar to that of Pt/CoPt-2/MWCNTs. As clearly seen from the TEM images and associated insets (Fig. 7b~d), CuPt (404), NiPt (111) and FePt (111) NPs have well-defined and uniformly spaced lattice fringes of 0.189 nm, 0.218 nm and 0.219 nm, respectively. Figure S6 obviously clarifies the elemental mapping of Pt and transition metals. These results demonstrate the homogenous distribution of Pt and other transition metal atoms in their alloyed NPs. Our facile synthesis method introduces a general and feasible route for the synthesis of binary alloys.

## Discussion

We proposed a novel route for the more efficient fabrication of MWCNT-supported Pt/CoPt composite NPs by solution plasma sputtering with subsequent thermal annealing. Pt/CoPt composite NPs were formed directly from metal wire electrodes without any additions, which contrasts with general wet synthesis methods. The as-synthesized Pt/CoPt-2/MWCNTs exhibited superior electrochemical activity for methanol oxidation in acidic media with a mass activity of 1719 mA mg^−1^_Pt_, which is much higher than that of previous reports of Pt-Co alloy and commercial Pt/C (3.16 times) because of the many active sites and clean catalyst surface. In addition, the Pt/CoPt-2/MWCNTs catalysts showed good stability towards methanol oxidation, which is attributed to the special synergistic effects of CoPt alloy. Moreover, our facile solution plasma sputtering-assisted synthesis method, which introduces a general and feasible route for the synthesis of different metals and binary alloys, can not only be applied to electro-catalytic systems but also offers potential applications in many fields.

## Methods

### Materials

Raw materials of platinum and transition metals (Co, Fe, Ni and Cu) wires with the diameters of 1.0030 mm (Aldrich, 99.9%) were used as opposite electrodes. Super-pure water was selected as a solvent medium. Multi-walled carbon nanotubes (MWCNTs, Mitsubish chemical Co.) were used as support materials.

### Materials Characterization

Phase compositions of all products were analysed by powder X-ray diffraction (XRD, Rigaka Smartlab) with Cu Kα (λ = 1.5418 Å) incident radiation at 30 kV voltage and 40 mA current. XRD patterns were recorded from 5 to 85 ° (2θ) with a scanning step of 8 °/min. The chemical composition and atomic structure of Pt-Co catalyst NPs were thoroughly characterized by transmission electron microscopy (TEM, JEM-2100F). X-ray photoelectron spectroscopy (XPS) measurements were performed with an AXIS UltraDLD spectrometer with a monochromatic Al KαX-ray source (1486.6 eV photos). The binding energy was calibrated by means of the C 1 s peak energy of 284.6 eV. The metallic composition of each catalyst was measured by inductively coupled plasma-mass spectrometry (ICP-MS, ICP OPTMA20000V).

### Electrochemical measurements

Electrochemical measurements were performed using a three-electrode system consisting of a glassy carbon working electrode (GCE, ϕ = 3 mm, geometrical area of 0.07 cm^2^), a platinum sheet counter electrode (1 * 1 cm^2^), and an Ag/AgCl reference electrode (saturated with 3.5 M aqueous KCl). The working electrode was prepared as follows: a 2-mL water suspension solution, including 5 mg of catalyst particles, was obtained by ultrasonic dispersion for 1 h with 10 μL of 5% aqueous Nafion. 6 μL of well-mixed water suspension was dispensed onto the surface of a clean glassy carbon electrode and dried at room temperature. A solution of 0.5 M H_2_SO_4_ purged with nitrogen gas for 40 min to remove dissolved oxygen was used as the electrolyte for the cyclic voltammetry (CV) measurements. The CV measurements were carried out within the potential range of −0.205–1.2 V in a solution of 0.5 M H_2_SO_4_ (N_2_ saturated) with a scan rate of 50 mV s^−1^ and used for the determination of the ECSA of Pt. The catalytic performance of the different catalysts in the room temperature MOR was also measured by cyclic voltammetry. The potential window of 0 V to 1 V was scanned at a rate of 50 mV s^−1^. The electrolyte was 0.5 M H_2_SO_4_ and 1 M MeOH, and the current density was normalized to obtain the mass activity and specific activity. Choronoamperometric curves were measured at a potential of 0.6 V for 5000 s.

### Data availability

The data that support the findings of this study are available from the corresponding author upon request.

## Additional Information

**How to cite this article:** Huang, H. *et al*. Facile Fabrication of Platinum-Cobalt Alloy Nanoparticles with Enhanced Electrocatalytic Activity for a Methanol Oxidation Reaction. *Sci. Rep.* **7**, 45555; doi: 10.1038/srep45555 (2017).

**Publisher's note:** Springer Nature remains neutral with regard to jurisdictional claims in published maps and institutional affiliations.

## Supplementary Material

Supplementary Information

## Figures and Tables

**Table 1:** Comparison analysis of methanol electro-oxidation activities of a few previous reports of Pt-based catalysts and our present results.

Pt based catalyst	ECSA (m^2^g^−1^)	mass activity (mA mg^−1^_Pt_)	specific activity (mA m^−2^_Pt_)	Ref
Pt−Co CMEs		135 mA with 0.5 mg cm^−2^ loading		43
Co_5_Pt_95_		1417	16	41
Pt–Co/PZAF-MWCNTs		231		44
3D Pt–Co networks	20.2	392.36	19.61	18
Pt_3_Co nanoflower	13.16	125.08	9.51	45
PtIrCo/3D carbon aerogel matrix	98 (20 mVs^−1^)	about 800 (20 mVs^−1^)		46
Pt-Co/CNTs	<80	<1300		47
Pt–Co	12.5	170		48
Pt-CoOx/MWCNTs	52.9	779.7		49
CoPt nanorods	195.4	909.9		50
Pt_15_Co_85_		509	16	51
hollow CoPt/MWCNTs	20.9	<250		52
commercial Pt/C	41.4	543	17.13	present work
Pt/MWCNTs	27.3	524	19.23	present work
Pt/CoPt-2/MWCNTs	83.7	1719	20.25	present work
