# Supplementary material for: Facile Fabrication of Platinum-Cobalt Alloy Nanoparticles with Enhanced Electrocatalytic Activity for a Methanol Oxidation Reaction
Source: Sci Rep. 2017 Mar 30;7:45555. doi: 10.1038/srep45555 (PMC5372086; doi:10.1038/srep45555)
Supplement: Supplementary Information [file srep45555-s1.doc]

Electronic Supplementary Materials

Facile Fabrication of Platinum-Cobalt Alloy Nanoparticles with Enhanced Electrocatalytic Activity for a Methanol Oxidation Reaction

Huihong Huang^123^, Xiulan Hu^123^*, Jianbo Zhang^123^, Nan Su^123^, JieXu Cheng^123^

^1^College of Materials Science and Engineering, Nanjing Tech University, Xin-Mo-Fan Road No. 5, 210009, Nanjing, Jiangsu, China

^2^The Synergetic Innovation Center for Advanced Material, y, Xin-Mo-Fan Road No. 5, 210009, Nanjing, Jiangsu, China

^3^Jiangsu National Synergetic Innovation Center for Advanced Materials (SICAM), Xin-Mo-Fan Road No. 5, 210009, Nanjing, Jiangsu, China

*Corresponding author at: College of Materials Science and Engineering, Nanjing Tech University, Xin-Mo-Fan Road No. 5, Nanjing, Jiangsu 210009, China.

Tel./fax: +86 25 8358 7260.

E-mail address: whoxiulan@163.com (X. Hu).


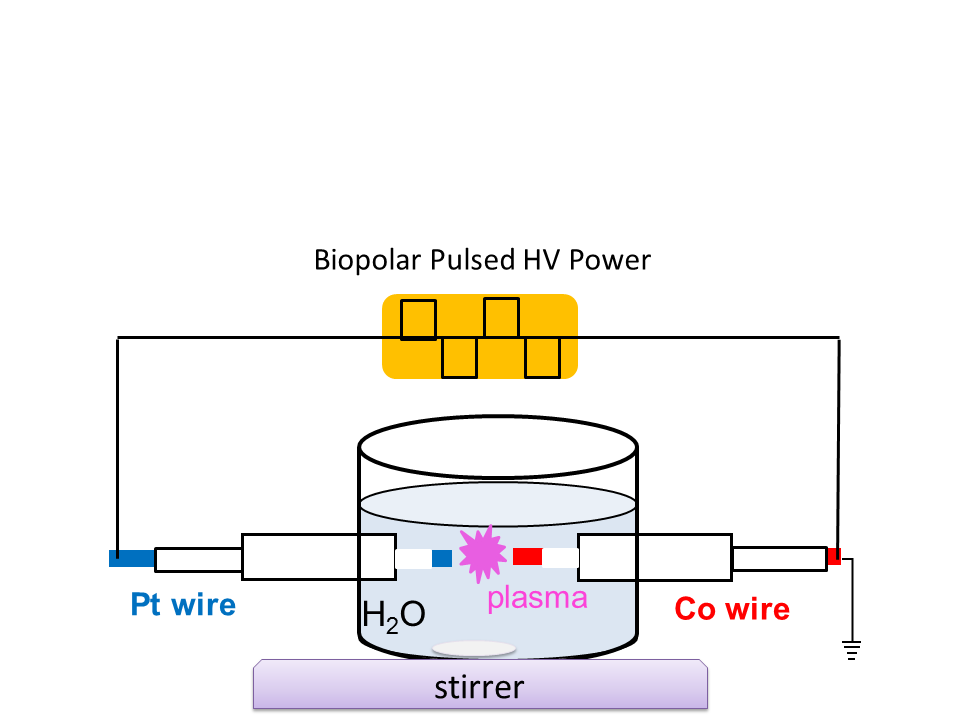


**Figure S1.** Schematic diagram of a solution plasma sputtering technique.


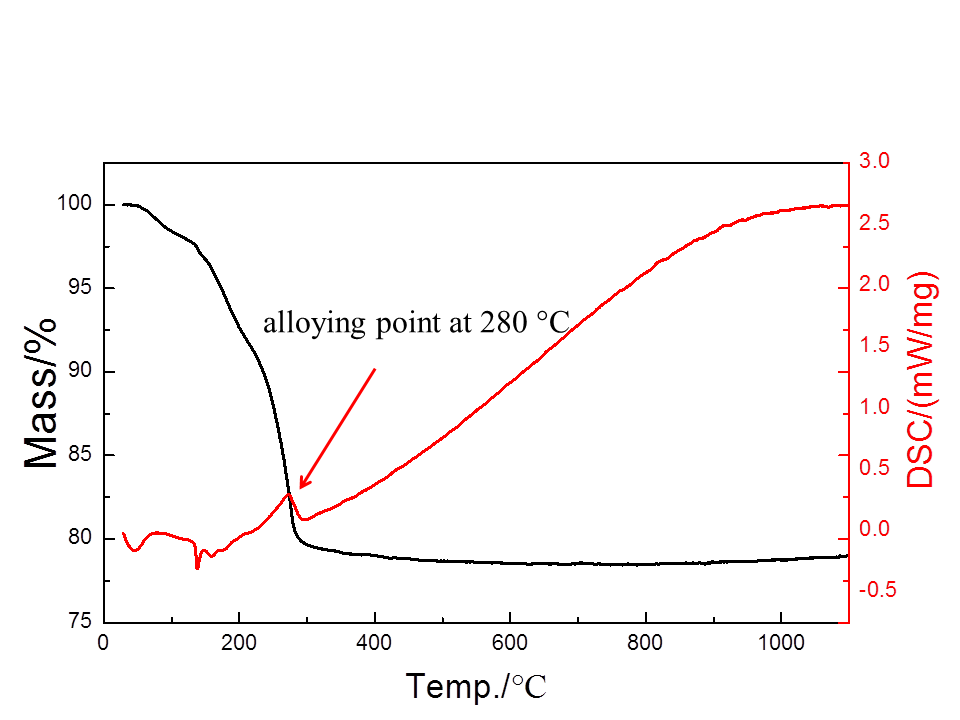


**Figure S2.** TG and DSC curves of CoPt alloy NPs.


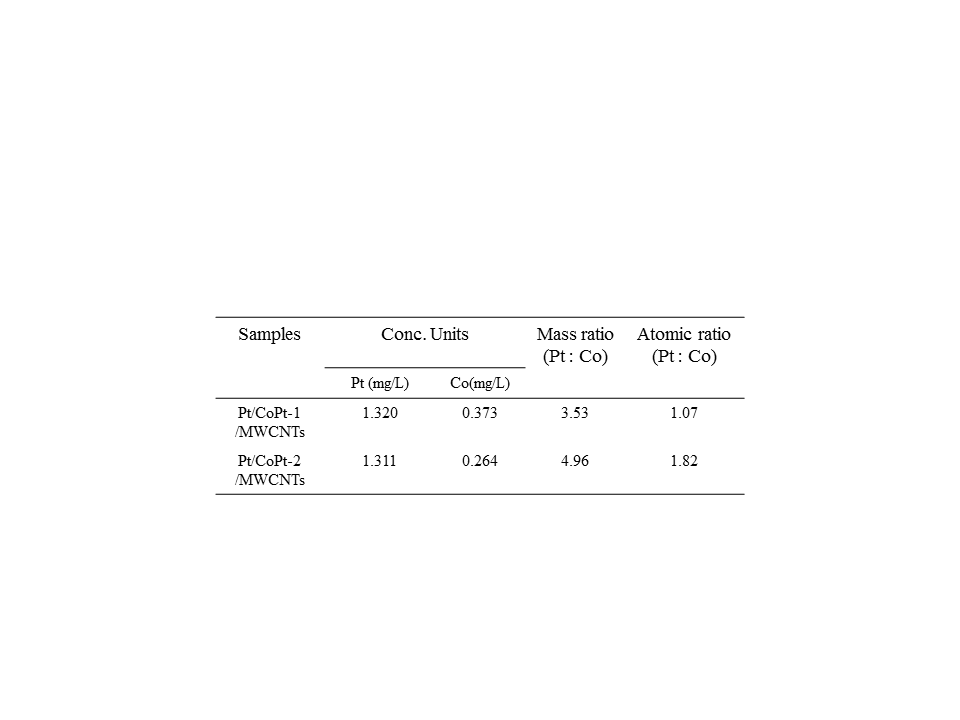


**Figure.** S3 The chemical composition of Pt–Co NPs measured by ICP-MS.


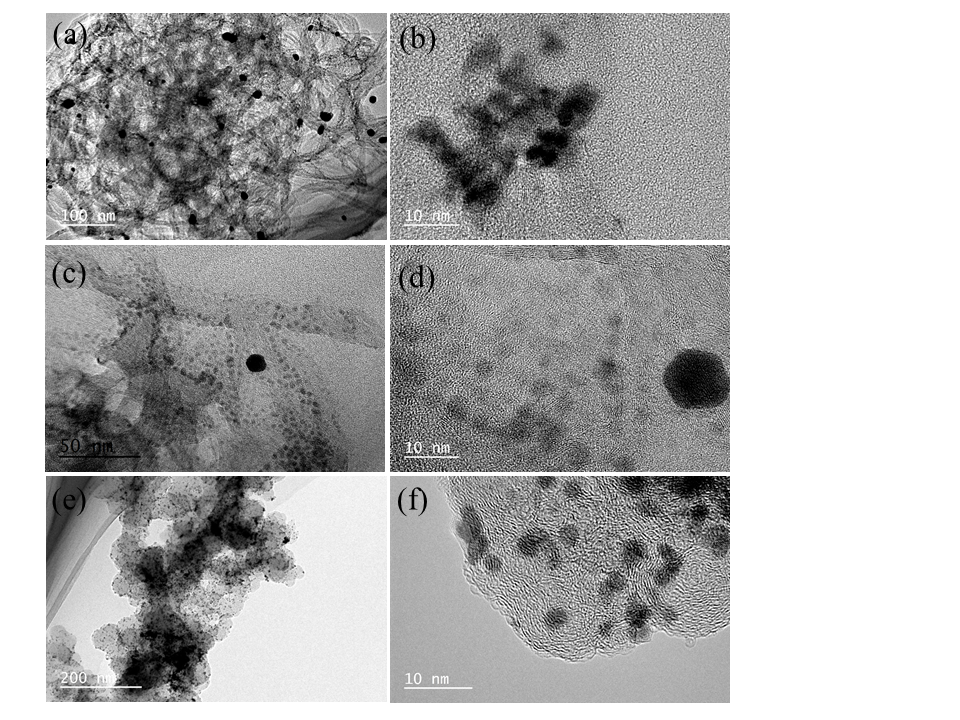


**Figure S4.** TEM and HR-TEM images of three catalysts. (a, b) Pt/CoPt-1/MWCNTs, (c, d) pure Pt/MWCNTs and (e, f) commercial Pt/C.


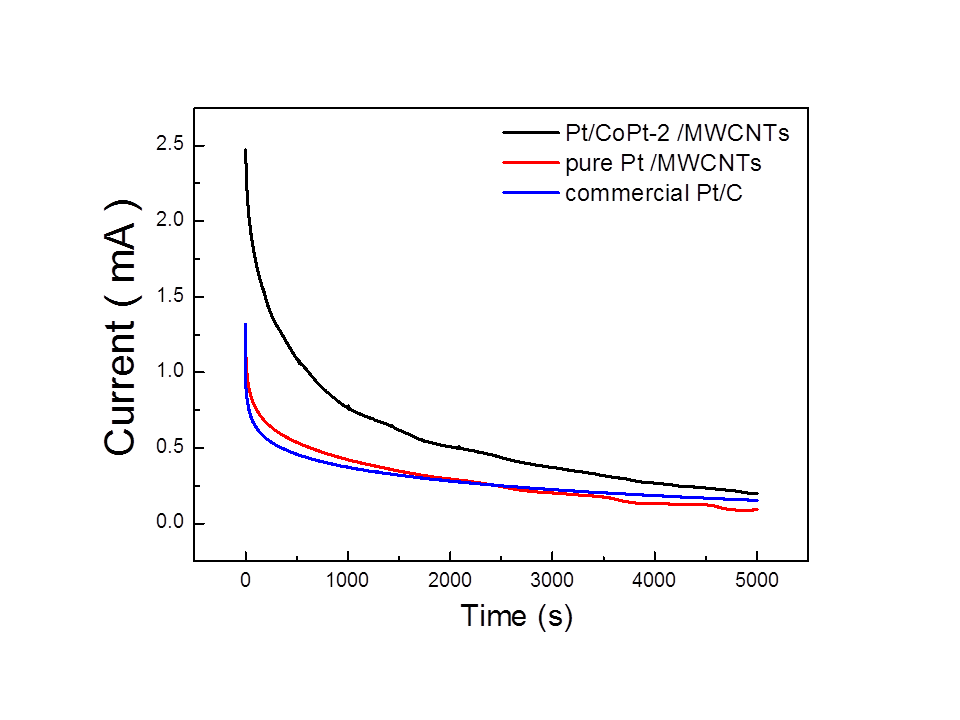


**Figure S5.** Choronoamperometric curves of three catalysts measured in 0.5 M H_2_SO_4_ and 1 M MeOH at a potential of 0.6 V for 5000 s.


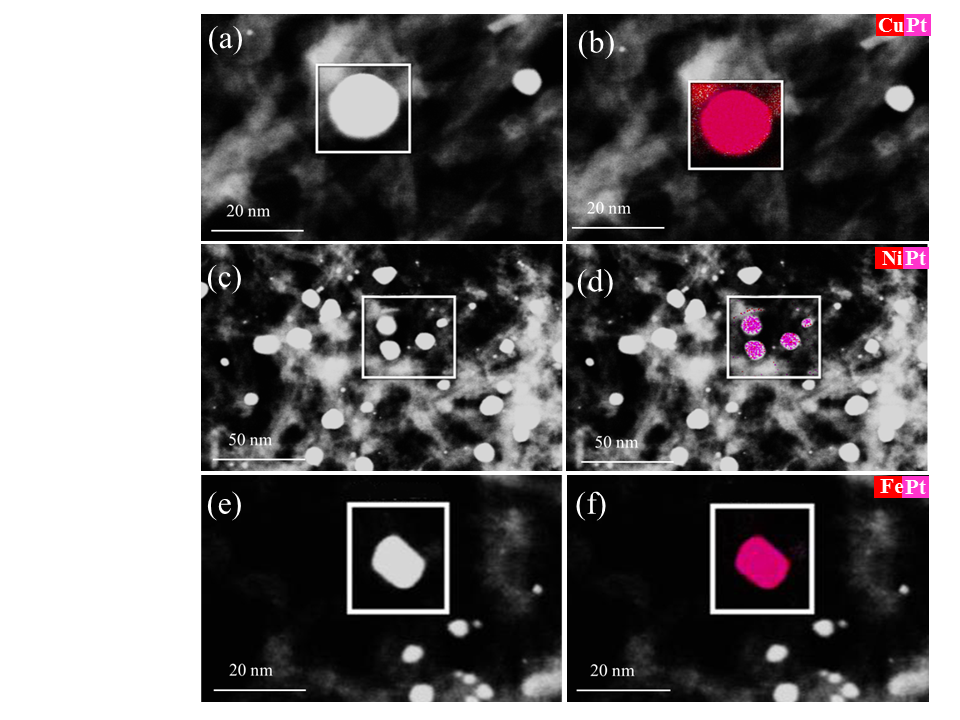


**Figure S6.** HAADF images and EDS mapping of Pt and M (M = Cu, Ni and Fe) in their alloys. (a - b) Pt/CuPt/MWCNTs, (c - d) Pt/NiPt/MWCNTs and (e - f) Pt/FePt/MWCNTs.
